# Origin and flow-mediated remodeling of the murine and human extraembryonic circulation systems

**DOI:** 10.3389/fphys.2024.1395006

**Published:** 2024-05-16

**Authors:** Kristof Van Schoor, Emmanuel Bruet, Elizabeth Anne Vincent Jones, Isabelle Migeotte

**Affiliations:** ^1^ Institut de Recherche Interdisciplinaire Jacques E. Dumont, Université Libre de Bruxelles (ULB), Brussels, Belgium; ^2^ Department of Cardiovascular Sciences, Centre for Molecular and Vascular Biology, Katholieke Universiteit Leuven (KU Leuven), Leuven, Belgium; ^3^ Department of Cardiology CARIM School for Cardiovascular Diseases Maastricht University, Maastricht, Netherlands

**Keywords:** embryo development, allantois, umbilical cord, yolk sac, vitelline vessels, placenta, mechanotransduction, blood flow

## Abstract

The transduction of mechanical stimuli produced by blood flow is an important regulator of vascular development. The vitelline and umbilico-placental circulations are extraembryonic vascular systems that are required for proper embryonic development in mammalian embryos. The morphogenesis of the extraembryonic vasculature and the cardiovascular system of the embryo are hemodynamically and molecularly connected. Here we provide an overview of the establishment of the murine and human vitelline and umbilico-placental vascular systems and how blood flow influences various steps in their development. A deeper comprehension of extraembryonic vessel development may aid the establishment of stem-cell based embryo models and provide novel insights to understanding pregnancy complications related to the umbilical cord and placenta.

## 1 Introduction

Mechanotransduction and vascular development are tightly linked together and the impact of mechanical stimuli on endothelial cells has been well documented (Campinho et al., 2020). Forces from blood flow affect endothelial cell polarization and migration, angiogenesis, lumen formation and vascular network remodeling. Three forces arise from blood flow: 1) shear stress, which is a consequence of shear flow, 2) circumferential stress, which is the force tangential to the vessel wall, and finally 3) axial stress, the force in the longitudinal axis of the vessel (Hoefer et al., 2013; Campinho et al., 2020). Shear stress is influenced by flow, viscosity, and vessel diameter, but it can also in turn affect the diameter of the vessel. Circumferential stress is regulated by blood pressure, vessel diameter and wall thickness and changes in circumferential stretch impact the thickness of the vessel wall. Axial stress is changed by longitudinal force, vessel diameter and wall thickness and will regulate vessel length (Hoefer et al., 2013). Mechanical stimuli from flow are sensed through several molecules including ion channels and cell-cell junctions (Table 1) (Rizzo et al., 1998; Tzima et al., 2005; Tarbell and Pahakis, 2006; Lucitti et al., 2007; Osol and Mandala, 2009; Li et al., 2014b; Li et al., 2014a; Garcia and Larina, 2014; Coon et al., 2015; Baeyens and Schwartz, 2016; Luu et al., 2018; Shin et al., 2019; Campinho et al., 2020; Daems et al., 2020; Mehta et al., 2020; Luse et al., 2023).

**TABLE 1:** Currently identified mechanosensors of blood flow forces in endothelial cells.

Mechanosensor	Function	References
Caveolae	Mechanosensation and rapid adaptation to changes in membrane tension	Rizzo et al. (1998)
		Luse et al. (2023)
		Shin et al. (2019)
Glycocalyx	Sensation and transmission of shear stress to the actin cytoskeleton and plasma membrane	Tarbell and Pahakis (2006)
Pecam1	Mechanosensory complex	Tzima et al. (2005)
Piezo1	Mechanosensitive non-selective cation channel	Li et al. (2014a)
Plexin D1	Mechanosensor	Mehta et al. (2020)
Primary cilia	Ca^2+^-dependent mechanosensors	Luu et al. (2018)
VE-Cadherin	Mechanosensory complex	Tzima et al. (2005)
VEGFR2	Mechanosensory complex	Shay-Salit et al. (2002)
		Tzima et al. (2005)
VEGFR3	Mechanosensory complex	Coon et al. (2015)

The vessels of the vitelline and umbilico-placental circulations are required for embryo development, as they provide nutrients and gas exchange. In addition, these extraembryonic vascular systems are functionally linked to the embryonic cardiovascular system (Linask et al., 2014). Vitelline vessels rely on embryonic circulation for their morphogenesis and remodeling (Jauniaux et al., 1991; Lucitti et al., 2007; Burton and Jauniaux, 2018a). As for placental vessels, the maternal uterine vasculature plays a large role in regulating hemodynamic stimuli (Osol and Mandala, 2009).

The yolk sac is conserved in many species and is the oldest extraembryonic structure present in vertebrates. Before the development of the placenta and the onset of the umbilico-placental circulation, the yolk sac provides nutrition to the embryo (Martinelli et al., 2023). The yolk sac consists of three layers: mesothelium, mesoderm, and endoderm. The way these three layers are oriented is everted between human and mouse, with the endoderm facing the endometrium in mice. While the yolk sac and placenta in mouse are not exactly like those in human, similar cell types are present with analogous functions. The endodermal layer provides nutrition to the embryo, whereas the mesodermal layer is at the origin of haemato- and angiogenesis (Rossant and Cross, 2001; Linask et al., 2014; Martinelli et al., 2023). The mouse yolk sac envelops the entire conceptus; the parietal yolk sac deteriorates around mid-gestation while the visceral yolk sac (formed of the three layers described above) persists until term. In human, the primary yolk sac will split in two: the smaller part deteriorates while the larger portion, the secondary yolk sac, remains until the end of the 20th week of pregnancy. Different to mouse, the human yolk sac does not envelop the conceptus but extends into the extraembryonic coelom (Martinelli et al., 2023).

The allantois is the precursor for the umbilical cord in mouse (Inman and Downs, 2007; Arora and Papaioannou, 2012) and contributes to the umbilical cord in human (Spurway et al., 2012; Basta and Lipsett, 2023). The murine allantois is capable of developing its vasculature independently and can be isolated and cultured *ex vivo* or transplanted to study vascular development and chorio-allantoic fusion (Downs and Harmann, 1997; Diehl et al., 2001; Downs et al., 2001; Downs, 2006; Arora and Papaioannou, 2012; Hou et al., 2016; Hadamek et al., 2018). In human, the umbilical cord contains one umbilical vein and two umbilical arteries (Spurway et al., 2012; Ramesh et al., 2015; Li et al., 2019; Ebbing et al., 2020), whereas the umbilical cord in mouse has single umbilical artery and vein (Inman and Downs, 2007). In human, the presence of only one umbilical artery may be associated with pregnancy and postnatal complications (Ramesh et al., 2015; Li et al., 2019; Ebbing et al., 2020).

The mesoderm layer of the yolk sac is the site where the first endothelial and blood cells form, in structures known as the blood islands, around embryonic day (E) 7 in mouse embryos (Lucitti et al., 2007; Linask et al., 2014; Martinelli et al., 2023). Primitive erythroblasts from the yolk sac enter the circulation after the heart starts beating, around E8 in mouse embryos (Lucitti et al., 2007). Live imaging data showed that endothelial cells originating from the yolk sac contribute to the endocardium, head vasculature and dorsal aortae in the mouse embryo (Collart et al., 2021), thereby confirming that the vitelline and embryonic vascular systems share cells of a similar origin. Aside from this cellular link, the extraembryonic vasculatures and the fetal cardiovascular system are also hemodynamically connected (Lucitti et al., 2007; Garcia and Larina, 2014; Linask et al., 2014; Burton and Jauniaux, 2018a; 2018b; Jauniaux et al., 2020). These connections will be the focus of this review, based on experimental data in mouse and findings from rare samples as well as functional analysis of pregnancies in human.

## 2 Mechanotransduction in the murine vitelline and umbilico-placental vasculatures

### 2.1 Vitelline vasculature

#### 2.1.1 From blood islands to vessels

**FIGURE 1:**
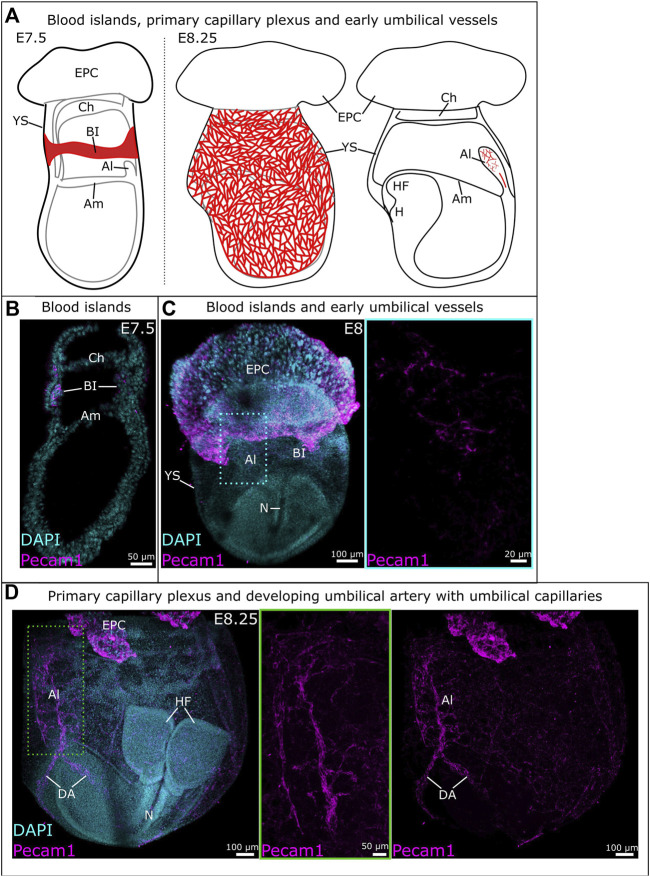
Development of vitelline and umbilical vessels between E7.5 and E8.25. **(A)** Schematic overview of vitelline vascular development from blood islands at E7.5 (left) to the primary capillary plexus at E8.25 (middle). Schematic representation of early umbilical vascular development at E8.25 (right). **(B)** Sagittal cryosection of an E7.5 mouse embryo. Blood islands were identified through immunofluorescent staining for Pecam1. **(C)** Whole mount E8 mouse embryo with vessels identified through Pecam1 staining. The blue frame provides a close-up of the umbilical vasculature. **(D)** Whole mount E8.25 mouse embryo with the umbilical vasculature (middle) and primary capillary plexus (right). A close-up of the umbilical vessels is provided in the green frame where the z-slices containing the vitelline vessels have been removed. Al: allantois; Am: amnion; BI: blood islands; Ch: chorion; DA: dorsal aorta; EPC: ectoplacental cone; H: heart; HF: headfold; N: notochord; YS: yolk sac.

The blood islands arise around E7-E7.5 in the proximal yolk sac (Figures 1A, B); they consist of endothelial (angioblasts) and hematopoietic progenitors. The first vessels are formed through vasculogenesis, prior to the start of hemodynamic forces (Lucitti et al., 2007; Garcia and Larina, 2014; Linask et al., 2014; Martinelli et al., 2023). Outer angioblasts proliferate and differentiate into endothelial cells that migrate distally into the yolk sac and organize into a rudimentary network of vessels (Ferkowicz et al., 2003). This network is known as the primary capillary plexus and is fully established around E8.5 (Figures 1A, C) (Udan et al., 2013a; Garcia and Larina, 2014). Around this same time (E8-E8.5), the embryonic heart starts beating, which triggers blood flow and thereby hemodynamic forces (Lucitti et al., 2007; Garcia and Larina, 2014; Linask et al., 2014).

**FIGURE 2:**
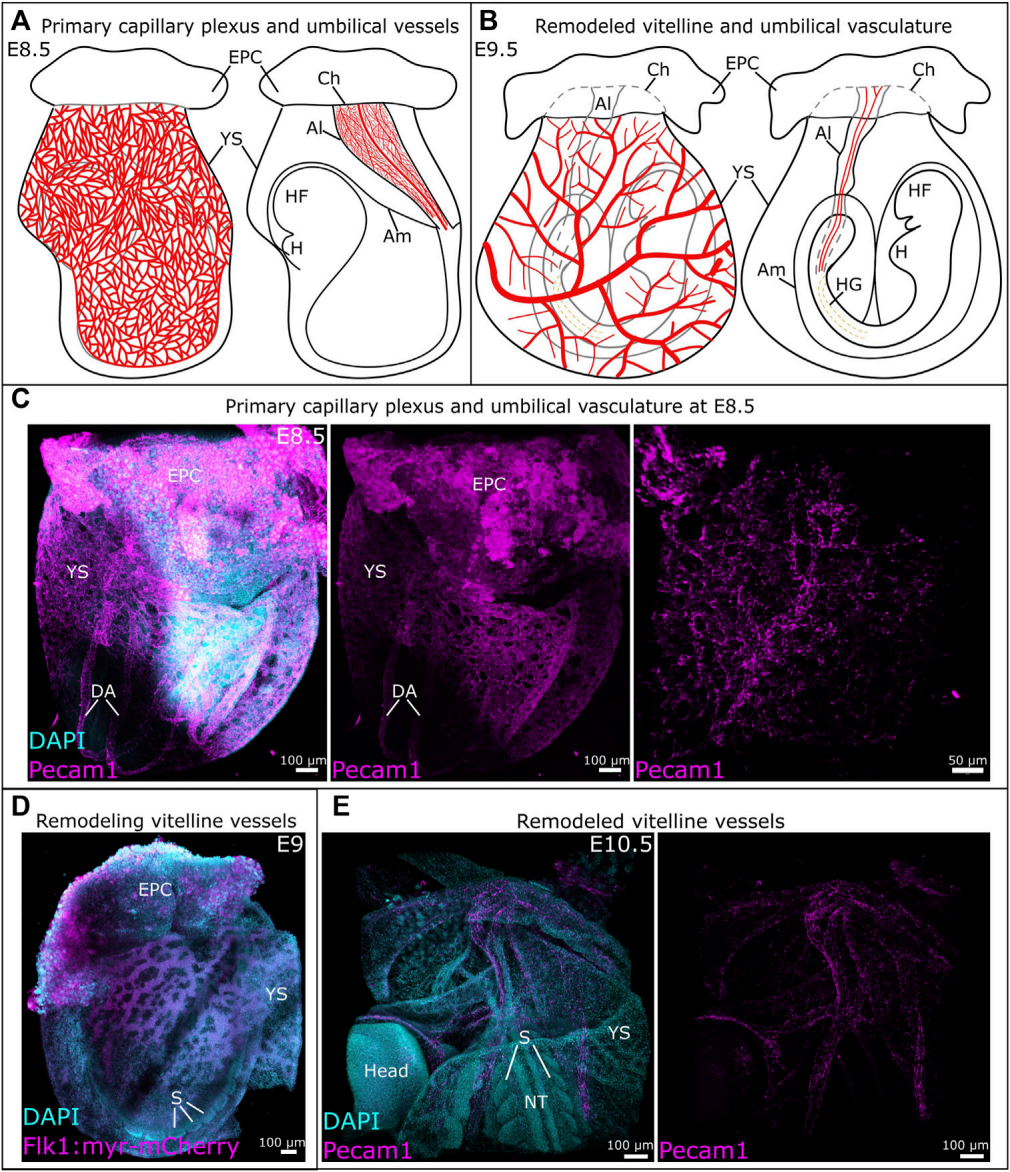
Development of vitelline and umbilical vessels between E8.5 and E10.5. **(A)** Schematic representation of the primary capillary plexus (left) and umbilical vessels (right) at E8.5. **(B)** Schematic overview of the remodeled vitelline vasculature (left) and umbilical vessels (right). **(C)** Whole mount E8.5 mouse embryo with vessels identified by Pecam1. A detailed view of the umbilical vessels is provided in the image on the right. **(D)** The remodeling vitelline vasculature in a Tg (Flk1::myr-mCherry) mouse embryo at E9. **(E)** The remodeled vitelline vascular system at E10.5, vessels are visualized through Pecam1. Al, allantois; Am, amnion; Ch, chorion; DA, dorsal aorta; EPC, ectoplacental cone; H, heart; HF, headfold; HG, hindgut; NT, neural tube; S, somite; YS, yolk sac.

Between E8.5 and E9.5, the vitelline vasculature remodels under the influence of mechanical stimuli originating from blood flow and circulating primitive erythroblasts (Lucitti et al., 2007). The remodeled vasculature of the yolk sac consists of a structured, hierarchical network of vessels with functional arteries and veins (Figures 2B, D, E) (Garcia and Larina, 2014). This remodeling is essential to the survival of the embryo, as demonstrated by the phenotype of several genetically modified mouse lines (Table 2). For example, impairment of TGFβ (Carvalho et al., 2007) or Notch (Krebs et al., 2004) signaling prevent vitelline vasculature remodeling and result in embryonic death at E10.5. Yolk sac vessels in Neuropilin-1 knockout embryos have a large diameter and fail to remodel (Jones et al., 2008). Deletion of Piezo1 impairs mechanosensation, which results in a failure to rearrange the network and ultimately embryonic death (Li et al., 2014a). In Foxo1^−/−^ embryos, even though blood flow is normal, failure to remodel leads to embryonic death by E10.5 (Li-Villarreal et al., 2022).

**TABLE 2:** Summary of experimental models showing failure to remodel the vitelline vasculature.

Protein/molecule	Model	Findings	References
Foxo1	Mouse embryo	KO results in failed vascular remodeling and embryonic death by E10.5	Li-Villarreal et al. (2022)
		Arterial fate specification through repression of *Sprouty2* and *Sprouty4*	
		Maintains Vegfr2 expression on arterial endothelial cells	
Mlc2a	Mouse embryo	KO results in cardiac defects and failed remodeling of the primary capillary plexus, resulting in embryonic death	Huang et al. (2003)
Notch	Mouse embryo	Impairment prevents vitelline vascular remodeling and results in embryonic death by E10.5	Krebs et al. (2004)
TGFβ	Mouse embryo	Impairment prevents vitelline vascular remodeling and results in embryonic death by E10.5	Jones et al. (2004)
			Carvalho et al. (2007)

#### 2.1.2 Blood flow forces drive the remodeling of the primary capillary plexus to a mature vascular system

Circulation of primitive erythroblasts from the yolk sac starts around E8.25, but their distribution in the vitelline and embryo circulation is uneven until E10. The hematopoietic progenitors are also enriched in the yolk sac until E10.5. Those observations show that the yolk sac vasculature remains a site of progenitor production and preferential adhesion even after the liver becomes a site of hematopoiesis (McGrath et al., 2003; Jones et al., 2004). A fully functioning heart creating sufficient pulsatile flow is required to drive vascular remodeling in the yolk sac. Embryos defective for myosin light chain 2a (Mlc2a) did not remodel the vitelline vessels because of reduced laminar flow (Lucitti et al., 2007). *Mlc2a* ^*−/−*^ embryos exhibited defects in atrial contraction and ventricular filling, resulting in slow and delayed blood circulation, and limited oscillatory movement of primitive erythroblasts. Although mutant embryos appeared normal at E8.5, the primary capillary plexus did not remodel by E9.5, so that their yolk sacs lacked both small and large vessels (Table 2) (Huang et al., 2003; Lucitti et al., 2007). Immobilizing the erythroblasts in the blood islands also prevented vascular remodeling and delayed embryo development. By artificially increasing the blood viscosity, without restoring erythroblast mobility, vascular remodeling and normal embryo development up to the axial rotation or turning stage (between E8 and E9) could be rescued (Lucitti et al., 2007).

Hematocrit levels influence laminar shear stress and correlate to vessel size, according to the Fåhræus-Lindqvist effect (Fåhræus and Lindqvist, 1931; Jones et al., 2004; Farina et al., 2021). In addition, laminar shear stress impacts various molecules that are known to be involved in development. For instance, laminar shear stress will induce platelet-derived growth factor A and B receptors, that are required for vessel stabilization via pericytes and mural cells (Jones et al., 2004). Furthermore, laminar shear stress can induce TGFβ signaling, which is required for the remodeling of the primary capillary plexus (Jones et al., 2004; Carvalho et al., 2007). Vegfr2, which is required prior to the appearance of flow for the formation of blood islands and the primary capillary plexus (Shalaby et al., 1995; Jones et al., 2004), is also a component of the mechanotransduction cascade (Shay-Salit et al., 2002; Lucitti et al., 2007; Osol and Mandala, 2009; Garcia and Larina, 2014; Campinho et al., 2020). *In vitro,* Vegfr2 was shown to be activated by laminar flow (Shay-Salit et al., 2002; Baeyens and Schwartz, 2016). At E8.5, prior to remodeling, the levels of laminar shear stress vary between vessels of similar size in the yolk sac. Between E9.5 and E10.5, similar sized vessels possess similar shear stress levels (Jones et al., 2004). These data correlate with earlier findings showing that a steady-state hematocrit is not reached before E10 and changes in the distribution of primitive erythroblasts are temporally regulated (McGrath et al., 2003).

#### 2.1.3 Piezo1 as a sensor for shear stress in endothelial cells

While it is well established that blood circulation is required to generate the hemodynamic forces that drive vascular remodeling (Lucitti et al., 2007), the exact mechanism by which endothelial cells sense these forces has long remained an enigma. In addition to adherens junctions (Shay-Salit et al., 2002; Lucitti et al., 2007; Osol and Mandala, 2009; Garcia and Larina, 2014; Baeyens and Schwartz, 2016; Campinho et al., 2020), the role of mechanically sensitive Ca^2+^ cation channels is becoming increasingly clear, with Piezo1 being the most studied in vascular development. Piezo proteins are subunits and assemble in trimers to form the functional mechanically sensitive channels (Ge et al., 2015; Guo and MacKinnon, 2017; Saotome et al., 2018; Zhao et al., 2018). Global or endothelial cell-specific disruption of Piezo1 reduced the Ca^2+^-entry evoked by shear stress, which resulted in vascular defects in the mouse yolk sac and embryonic lethality at E9.5 (Li et al., 2014a; Ranade et al., 2014), with a minority of embryos surviving up to E16.5 (Li et al., 2014a). Piezo1 complete or partial deletion reduced the number of large vessels in the vitelline vasculature (Li et al., 2014a; Ranade et al., 2014). Endothelial cells were organized in a cobblestone pattern in *Piezo1* ^*+/−*^ embryos at E9.5, whereas they had a linear appearance in *Piezo1* ^*+/+*^ control littermates, indicating that haploinsufficiency reduced the capacity of endothelial cells to align in the direction of flow (Li et al., 2014a). Calpain-2 impacts the actin cytoskeleton and focal adhesions and has also been suggested to be involved in endothelial cell alignment under shear stress (Lebart and Benyamin, 2006; Miyazaki et al., 2007; McHugh et al., 2010; Li et al., 2014a). Calpain-2 activity was significantly reduced in *Piezo1* ^*−/−*^ embryos compared to controls. Proteomic analysis confirmed that Piezo1 regulates the calpain-2 system through Ca^2+^-influx. Therefore, these experiments suggest that Piezo1 is required for shear stress sensing and regulates endothelial cell alignment to blood flow via the calpain-2 system (Li et al., 2014a). Deletion of *Capn4*, which encodes the regulatory subunit of Calpains, leads to embryonic lethality at E10.5, attributed to heart defects (Arthur et al., 2000). It is possible that disrupted vascular development in the yolk sac might play a role in embryo arrest in that model as well.

#### 2.1.4 A common key transcription factor regulates arterial fate specification, mechano-sensing, and vascular remodeling in the yolk sac

Arterial and venous identities in the yolk sac are, to some extent, already established before blood flow starts (Herzog et al., 2005; Aitsebaomo et al., 2008; Chong et al., 2011; Li-Villarreal et al., 2022). However, arterio-venous specification remains somewhat ambiguous and plastic, and its completion is dependent on hemodynamical forces and blood flow (le Noble et al., 2004; le Noble et al., 2005; Jones et al., 2006; Wragg et al., 2014; Li-Villarreal et al., 2022).

The transcription factor Forkhead box protein O1 (Foxo1) is required for pre-flow arterial specification and subsequent vascular remodeling, specifically in the yolk sac. Embryos deficient for *Foxo1* in endothelial cells (*Foxo1* ^*ECKO*^) are indistinguishable from wild type until E8.5, including blood flow velocity. At E9.5 the vitelline vessels fail to remodel, blood flow velocity has now slowed compared to the WT littermates, and mutant embryos show symptoms of heart failure, leading to embryonic lethality around E11.5. It is likely that the decrease in flow velocity in the mutants is a consequence of the heart beating against an immature vitelline network (Table 2) (Li-Villarreal et al., 2022). Germline defects in *Foxo1* display a similar phenotype, plus additional defects in allantois and chorio-allantoic fusion with partial penetrance (Ferdous et al., 2011; Li-Villarreal et al., 2022). Mechanistically, deep analysis of the mutant phenotype showed that *Foxo1*-mediated repression of *Sprouty2* and *Sprouty4* specifically in the yolk sac is necessary to maintain an arterial gene expression profile (notably *Dll4*) as well as the expression of *Vegfr2* (Li-Villarreal et al., 2022).

Remodeling of the vitelline vasculature relies on endothelial cell migration and fusion of vessels (Udan et al., 2013b; Campinho et al., 2020; Li-Villarreal et al., 2022). While arterial endothelial cells in the yolk sac showed directional migration to the hemodynamic stimuli, those under lower flow and those in the vitelline veins migrated in a random manner (Udan et al., 2013b). The disturbance of Vegf-Vegfr2 signaling in *Foxo1* ^*ECKO*^ embryos might thus prevent arterial cells from properly responding to mechanical signals. Whether there is also a connection between the repression of arterial fate and failed remodeling requires further investigation. Thus, whether the defects in vascular remodeling in the yolk sac of *Foxo1* ^*ECKO*^ embryos are solely related to reduced Vegfr2 levels or also to the repression of arterial fate remains unclear (Li-Villarreal et al., 2022).

### 2.2 The murine umbilical cord and placenta

#### 2.2.1 From gastrulation to the umbilical cord

The allantois is the precursor for the umbilical cord. While it was thought to develop solely from primitive streak-derived extraembryonic mesoderm (Inman and Downs, 2007; Arora and Papaioannou, 2012; Downs, 2022), recent studies unveiled a possible input from streak-associated extraembryonic visceral endoderm through an epithelial-to-mesenchymal transition (Rodriguez and Downs, 2017; Downs and Rodriguez, 2020; Downs, 2022). The allantois first appears as a bud at the posterior embryonic-extraembryonic border, then extends into the exocoelomic cavity towards the chorion (Figure 1A). Between E8.25 and E8.75, the allantois fuses to the chorion, which initiates the development of fetus-derived vessels of the placental labyrinth (Figures 2A, B) (Watson and Cross, 2005; Arora and Papaioannou, 2012; Downs, 2022; Elmore et al., 2022).

Blood vessels in the allantois develop *de novo*, like in the yolk sac. However, in contrast with the yolk sac, vasculogenesis in the allantois is not accompanied by erythropoiesis (Downs et al., 1998). The first angioblasts appear in the distal portion of the allantois around E7.75-E8 (Figures 1A, C) and vasculogenesis takes place in a distal-to-proximal path (Downs et al., 1998; Downs, 2022). A branched vascular network develops distally while the umbilical artery forms without branches in the proximal third of the allantois (Figures 1A, C). At the base is the vessel of confluence, the site where the umbilical artery, omphalomesenteric artery and dorsal aorta meet. The unbranched nature of the developing umbilical artery is likely to ensure its proper connection to the vessel of confluence (Rodriguez and Downs, 2017; Rodriguez et al., 2017; Downs, 2022). Hedgehog is believed to be involved in the visceral endoderm epithelial-to-mesenchymal transition, and is required for the patterning of the arterial vessels of the allantois (Rodriguez and Downs, 2017).

#### 2.2.2 The maternal-fetal interface

Placentation is a crucial step in the development of mammalian embryos. In mice and men, defective placentation causes pregnancy pathologies going from intrauterine growth restriction to embryonic death (Rossant and Cross, 2001; Burton and Jauniaux, 2018b; Perez-Garcia et al., 2018; Woods et al., 2018; Lu et al., 2019). Hypoxia is a strong driving force for placenta formation but also a consequence of placental defects (Soares et al., 2017; 2018).

The labyrinth arises by folding of the chorion and its invasion by the allantois. It develops after chorio-allantoic fusion, around E8.5, and has a branched vasculature by E9. The mesoderm-derived allantois promotes the formation of three distinct trophoblast cell lineages from the chorion ectoderm: the cytotrophoblasts and syncytiotrophoblasts type I and type II. These three cell types form the transport layer across which nutrients and waste are exchanged. In addition, they line the maternal blood sinusoids, where blood enters from the spiral arteries and canals, and are juxtaposed to the endothelial cells from the fetal vessels (Rossant and Cross, 2001; Watson and Cross, 2005; Woods et al., 2018; Hemberger et al., 2020; Elmore et al., 2022). By E10, the embryo becomes reliant on the umbilico-placental vasculature for its survival (Woods et al., 2018).

In mouse, the ectoplacental cone, derived from polar trophectoderm, is a structure that resembles a cap on top of the extraembryonic ectoderm and separates the conceptus from the decidua. From the core of the ectoplacental cone, the junctional zone of the placenta will develop. The junctional zone consists of two layers, formed by three cell types. The outer layer contacts the decidua and contains parietal giant cells, whereas the inner layer is constituted by spongiotrophoblasts and glycogen trophoblasts (Rossant and Cross, 2001; Watson and Cross, 2005; Woods et al., 2018; Hemberger et al., 2020; Panja and Paria, 2021).

The uterine arteries branch at the implantation site, dividing into the spiral arteries (Panja and Paria, 2021). The outer cells of the ectoplacental cone differentiate into secondary trophoblast giant cells. These cells penetrate the endometrial stroma and connect to the maternal spiral arteries where they erode the smooth muscle cell layer and replace the endothelial cells, forming the blood canals through which maternal blood enters the maternal sinusoids in the labyrinth. Because trophoblast cells replace the endothelial cells of the maternal vessels, maternal blood in the placenta is in contact with fetal-derived cells instead of maternal ones (Woods et al., 2018; Panja and Paria, 2021). Interestingly, this contact between maternal blood and fetal-derived cells does not elicit an immune response.

The placenta presents an essential role in inducing an immune-privileged environment (Erlebacher, 2013; Woods et al., 2018) at the maternal-fetal interface, through the regulation of maternal immune cells populating the decidua. These immune cells are highly specialized, avoiding any placental attack as a foreign organ transplant and protecting the embryo from infection. Specifically in the first trimester, the decidua is primarily populated by natural killer cells (∼70%) and macrophages (∼20%) in both human and mouse. In human, decidual natural killer cells first appear in the secretory endometrium prior to implantation and express high levels of chemokines and cytokines. In mouse however, natural killer cells only appear during pregnancy. These decidual natural killer cells play a major role in spiral arteriole remodeling to maximize maternal blood flow through the placenta (Chazara et al., 2011; Zhang et al., 2011). Interestingly, in human the cytolytic function of decidual natural killer cells is inhibited through the expression of non-classical class I molecules (i.e., HLA-E and HLA-G) expressed by extravillous trophoblasts. In addition, decidual natural killer cells express IL-10, which induces the differentiation of decidual macrophages and maintains a non-inflammatory state. Similarly to natural killer cells, decidual macrophages contribute to spiral arteriole remodeling and both cells express IL-15.

#### 2.2.3 Hemodynamics in the umbilical cord and placenta throughout gestation

In 2006, Mu and Adamson measured blood flow in the mouse umbilical and placental arteries, using Doppler ultrasound (Table 3). As gestation progresses, the resistance to blood flow in the uterine arteries decreases and blood is allowed to flow more freely, as was indicated by an increase in peak systolic and end-diastolic velocities. Blood flow was detected in the maternal arterial canal from E10.5 on, and in its branches starting from E12.5. While initially slow, flow velocity increased over time. In the spiral arteries however, blood flow was not always detected (Bernstein et al., 2002; Mu and Adamson, 2006). The slow pace of blood movement within the placenta helps facilitate adequate exchange of nutrients and waste between the maternal and fetal blood (Linask et al., 2014).

**TABLE 3:** Overview of the evolution of blood flow velocity throughout development.

Model	Tissue	Developmental stage	Velocity	References
Mouse	Uterine artery	Not pregnant	23 cm/s^a^	Mu and Adamson (2006)
	Uterine artery	E15.5	52 cm/s^a^	Mu and Adamson (2006)
	Uterine artery	E18.5	60 cm/s^a^	Mu and Adamson (2006)
	Umbilical artery	E14.5	10 cm/s^a^	Mu and Adamson (2006)
	Umbilical artery	E18.5	15 cm/s^a^	Mu and Adamson (2006)
	Ductus venosus	E17.5	14.65–17.71 cm/s^a^	Zhou et al. (2014)
	Intrahepatic umbilical vein	E17.5	4.95–5.93 cm/s^c^	Zhou et al. (2014)
Human	Uterine artery	Not pregnant	32–44 cm/s^a^	Bernstein et al. (2002)
				Mu and Adamson (2006)
	Uterine artery	18–20 weeks	70–130 cm/s^a^	Mu and Adamson (2006)
	Umbilical artery	18–20 weeks	27 cm/s^a^	Mu and Adamson (2006)
	Umbilical artery	38–40 weeks	36 cm/s^a^	Mu and Adamson (2006)
	Umbilical vein	20 weeks	63 mL/min^b^	Barbieri et al. (2023)
	Umbilical vein	38 weeks	373 mL/min^b^	Barbieri et al. (2023)

^a^ Peak velocity.

^b^ Absolute flow volume.

^c^ Time-average maximal velocity over whole cardiac cycle.

At late E8.5, blood flow becomes detectable in the umbilical artery. Flow velocity in the umbilical artery increases during gestation and is paired with a decrease in vascular resistance. In the umbilical veins, pulsatile flow was also detected. However, these pulsations likely originate from retrograde waves that are caused by the contractions of the heart (Mu and Adamson, 2006).

#### 2.2.4 Remodeling of the umbilical vasculature

Piezo1 acts as a mechanosensor in the vitelline vasculature. It was also demonstrated *in vitro* that endothelial cells derived from human umbilical vein respond to shear stress through Piezo1 (Li et al., 2014a). Therefore, a similar role for Piezo1 in the murine umbilical vasculature seems not too farfetched. In addition, umbilical endothelial cells express Vegfr2, Pecam1, VE-cadherin and eNOS. These are involved in angio- and vasculogenesis, as well as the normal functioning of blood vessels, and are also involved in the mechanosensation of hemodynamic forces (Lucitti et al., 2007; Osol and Mandala, 2009; Garcia and Larina, 2014; Baeyens and Schwartz, 2016; Campinho et al., 2020).

Various *ex vivo* methods to study the allantois are available (Downs, 2006; Arora and Papaioannou, 2012; Hou et al., 2016; Hadamek et al., 2018). However, they do not readily provide a means to incorporate hemodynamic factors (Arora and Papaioannou, 2012). Blood flow in the umbilical cord only starts after chorio-allantoic fusion and after the umbilical artery has connected to the vessel of confluence (Linask et al., 2014). Perhaps it is because of these reasons that there is very little data available on the role of mechanotransduction in the development of the allantois and its vasculature in the mouse.

#### 2.2.5 Remodeling of the utero-placental vasculature

The uterine arteries must expand to support the fetoplacental unit after the placenta is formed and until birth. The lumen of the uterine arteries expands and doubles in size during gestation in mice. Interestingly, and as opposed to other species, the tunica media of the uterine arteries expands as well (van der Heijden et al., 2005; Osol and Mandala, 2009). The uterine arteries grow in the longitudinal axis too, approximately doubling their length in comparison to a non-pregnant state. Considering Hagen-Poiseuille’s law for laminar flow, extension of the artery increases its resistance to flow, whereas luminal expansion decreases this resistance. In case both parameters are doubled, resistance to flow would still decrease since length has a linear relationship to resistance while the radius has an inverse and quadratic relationship (Osol and Mandala, 2009). The uterine veins also expand (Forbes and Taku, 1975; Osol and Mandala, 2009).

Nitric oxide (NO) and eNOS are important factors in endothelial mechanosensation that can be influenced by progesterone and estrogen (Osol and Mandala, 2009). Estrogens stimulate vascular remodeling of the uterine arteries and invasion of the spiral arteries in humans, supporting an increased blood flow that is accompanied by an increase in shear stress. Estrogens drive NO and VEGF synthesis by ECs (Mandalà, 2020). In addition, *in vitro* experiments on human umbilical vein endothelial cells (HUVECs) showed that progesterone causes a rapid increase of NO production through the PI3K/Akt pathway (Pang et al., 2015). Knockout of *eNOS* impairs uterine artery growth during pregnancy. However, uterine arteries in pregnant mutant animals were still larger than those from non-pregnant females, suggesting that other factors are also at play (van der Heijden et al., 2005; Osol and Mandala, 2009).

## 3 Mechanotransduction in the human vitelline and umbilico-placental vasculatures

Similar to mouse, the human extraembryonic vasculature is comprised of two circulation circuits: 1) the vitelline circulation in the secondary yolk sac during early development, and 2) the umbilico-placental circulation towards the end of the first trimester (Burton and Jauniaux, 2018a).

### 3.1 Human vitelline vasculature

#### 3.1.1 Formation of the primary circulation system

**FIGURE 3:**
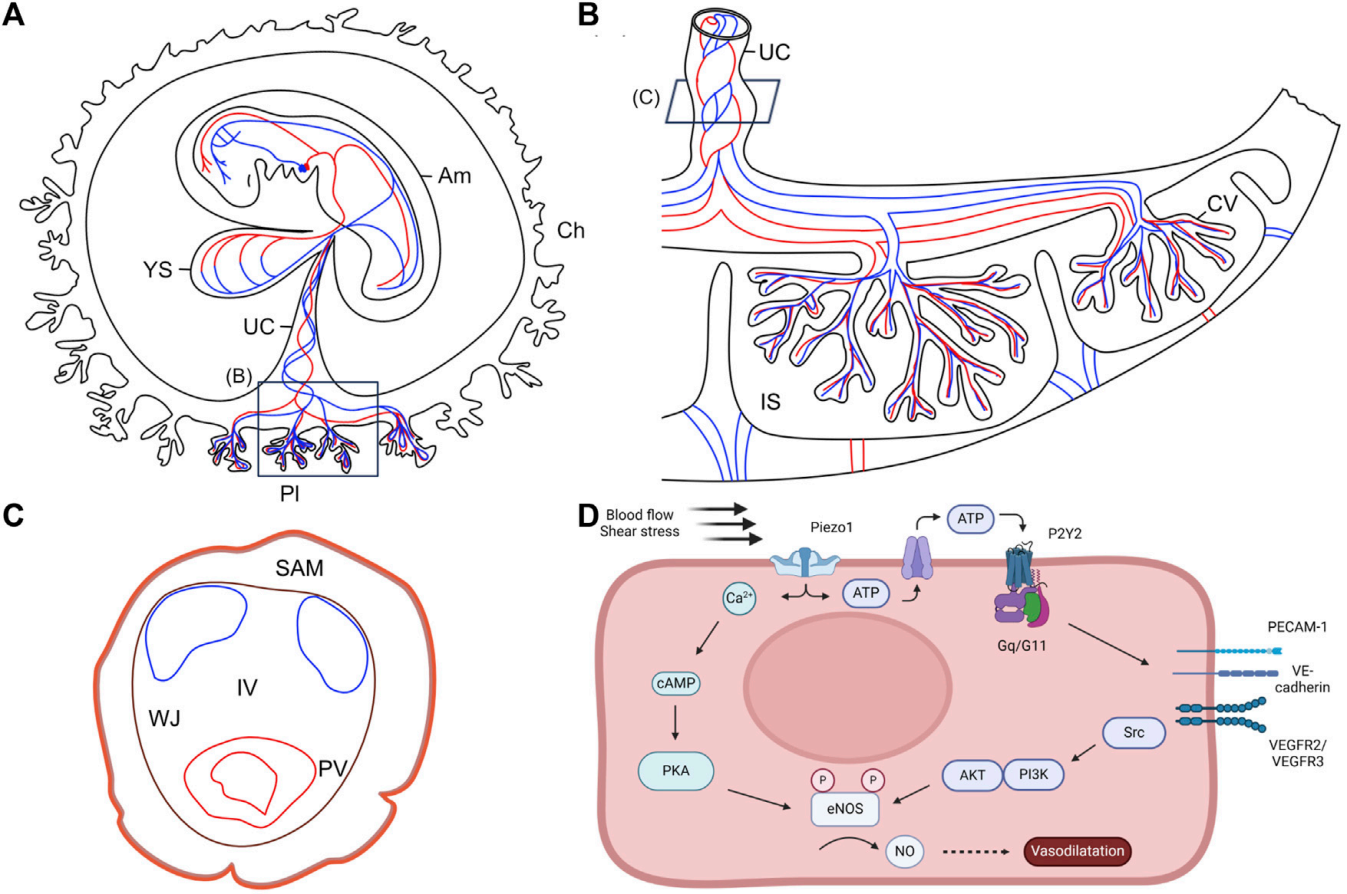
Anatomy of the human embryo and its extraembryonic structures. **(A)** Schematic representation of the human embryo and its extraembryonic structures during the fourth week of pregnancy. Adapted from Martinelli et al., 2023, with permission from SNCSC. **(B)** Schematic representation of the human term placenta. Based on Silini et al., 2020. **(C)** Schematic representation of the full-term human umbilical cord. Umbilical arteries are represented in blue and umbilical vein is represented in red. Based on Blanco-Elices et al., 2022. **(D)** Schematic illustrating the molecular pathways of vascular remodeling mediated by shear stress. AKT: protein kinase; Am: amnion; Ca^2+^: calcium ions; cAMP: cyclic adenosine monophosphate; ATP: adenosine triphosphate; Ch: chorion; CV: chorionic villi; eNOS: endothelial nitric oxide synthase; IS: intervillous space; IV: intervascular zone; NO: nitric oxide; P2Y2: purinergic receptor; PECAM-1: platelet endothelial cell adhesion molecule 1; PI3K: phosphatidylinositol 3-kinase; PKA: protein kinase A; Pl: placenta; PV: perivascular zone; SAM: subamnioblastic zone; UC: umbilical cord; V-cad: vascular endothelial cadherin; VEGFR: vascular endothelial growth factor receptor; WJ: Wharton’s jelly; YS: yolk sac.

In human, the yolk sac is a temporary organ, present only in early pregnancy. The human yolk sac was first described *in vivo* in 1979, as “a round, translucent, cyst-like structure” located in the exocoelomic cavity, by Mantoni and Pederson, using ultrasound scanning (Mantoni and Pedersen, 1979). Due to difficult access to early human embryos and the fragile structure of the yolk sac (Jauniaux et al., 1991), it has rarely been studied and its biological functions are relatively poorly understood (Gulbis et al., 1998; Burton and Jauniaux, 2018a). Nonetheless, it is well established that the yolk sac plays an essential role as the first circulatory system of the embryo. Starting during the second week of gestation (Jordan, 1910; Palis and Yoder, 2001; Tavian and Péault, 2005), the human yolk sac produces nucleated erythrocytes synthesizing hemoglobin (Luckett, 1978). Hematopoiesis takes place in extraembryonic mesoderm-derived cells that organize as blood islands in the mesenchymal layer of the secondary yolk sac. Most inner cells of the islands differentiate into blood cell progenitors, while peripheral cells become endothelial cells. Secondary to the start of heartbeats (around 3 somite stage) and the onset of blood flow (around 6 somite stage), blood islands angioblasts coalesce in a process called vasculogenesis to form the yolk sac primitive capillary plexus, which is then progressively remodeled. The primitive circulatory system is organized in a hierarchical network of large vessels ensuring high volumetric blood flow and small vessels carrying low flow. This vascular network connects the yolk sac to the embryo with a functional circulation via vitelline blood vessels between the yolk sac and the developing heart (Figure 3A). Until the placenta is sufficiently developed, this pre-portal system allows the transport of nutrients and O_2_ extracted from extraembryonic coelomic fluid to the embryonic tissues via vitelline veins and the elimination of metabolic waste and CO_2_ from the embryonic heart to the yolk sac via the vitelline artery (Martinelli et al., 2023). Therefore, aberrant formation of this primary circulation system could lead to growth retardation, cardiac failure, and embryonic death (Lucitti et al., 2007; Graupera et al., 2008; Culver and Dickinson, 2010).

### 3.2 Human umbilico-placental vasculature

#### 3.2.1 Development of the human umbilico-placental vasculature

The definitive placenta is formed by the end of the first trimester, concomitant to the degeneration of the yolk sac, after a series of extensive remodeling steps (Jauniaux et al., 1991; Burton and Jauniaux, 2018a). Firstly, villi develop in the entire gestational sac. After 8 weeks of gestation, the villi from the superficial pole of the placenta regress, leaving a smooth membrane called chorion laeve (Figure 3A). This regression occurs simultaneously with the entry of the maternal arterial circulation to the placenta, initially in the periphery and then in the entire placenta (Burton and Jauniaux, 2018a). It requires the migration of extravillous trophoblastic cells into the placental bed, and is modulated by high level of oxidative stress within the villi (Jauniaux et al., 2003). As the endothelial cells forming the capillaries in the regressing villi lose integrity, they become avascular. This regression is important to determine the final size and shape of the placenta (Figure 3B) (Burton et al., 2010). Indeed, an excessive or asymmetric recession of the villi can lead to ellipsoid placentas or eccentric insertion of the umbilical cord, which restrict nutrient supply to the fetus and impact the developing heart (Burton and Jauniaux, 2018a).

#### 3.2.2 Hemodynamic regulation in the human umbilical cord

Even though the precise molecular etiology and the direction of causality is often unclear, there is a clinical correlation between alterations of extraembryonic tissues and pregnancies characterized by intrauterine growth restriction and small babies at birth. For instance, cases of intrauterine growth restriction may be associated with an abnormal placenta, displaying immature or less abundant villi, presumably secondary to impaired branching angiogenesis and endothelial dysfunction, and creating a hypoxic environment (Kingdom et al., 2000).

Fluid mechanics and shear stress have a major impact on both the fetal cardio-vascular system and the umbilico-placental vasculature. In general, a high blood flow increases wall shear stress and the diameter of blood vessels (vasodilation), while a low blood flow reduces wall shear stress and vessels diameter (vasoconstriction) (Saw et al., 2018). While there seems to be no correlation between shear stress and diameter in the umbilical arteries, a strong relationship was found between shear stress and the diameter of the umbilical vein. A reduced velocity of blood flow in the umbilical vein and/or an impaired blood viscosity resulting in increased resistance to flow both result in the insufficient transfer of nutrients and oxygen to the fetus. Since blood vessels in the placenta lack autonomic innervation (Myatt, 1992), the regulation of vessel diameter relies on the production of vasoactive agents by endothelial cells, such as NO (Lu and Kassab, 2011), endothelin 1 (ET1) (Egorova et al., 2012), VEGF (dela Paz et al., 2012), or placental growth factor (PlGF) (Rashdan and Lloyd, 2015) to maintain a homeostatic level. These homeostasis mechanisms were only observed in umbilical arteries and seem to be absent or weak in umbilical vein, which could explain the lack of correlation between shear stress and umbilical arterial diameter (Saw et al., 2018).

The two umbilical arteries form helical structures as they coil around the umbilical vein. This helical geometry of the blood vessels in the umbilical cord is already visible at 7 weeks of gestation and appears important for the health of the pregnancy. Indeed, in about 2%–5% of human fetuses, an absence of blood vessel coiling, preventing hemodynamic changes, was observed and correlated with an increased incidence of fetal death (Strong et al., 1994; Saw et al., 2017).

The umbilical vessels are embedded in Wharton’s jelly, a protective mucous connective tissue, and contained within an outer layer of amnion (Figure 3C) (Spurway et al., 2012; Murphy et al., 2020). Degeneration or absence of Wharton’s jelly are risk factors for vessel compression, disruption of fetal blood flow, and eventually fetal hypoxia which can lead to fetal death (Kulkarni et al., 2007; Murphy et al., 2020). Wharton’s jelly degeneration is indicated by a decreased cord diameter in late gestation and is usually caused by a reduction of its water content. Absence of Wharton’s jelly is an extremely rare and severe disorder of pregnancy which results in intrauterine growth retardation, fetal morbidity, or perinatal death (Weissman et al., 1994; Spurway et al., 2012).

The two first cases of Wharton’s jelly degeneration were described in 1961 by Bergman et al. Analysis of the cords revealed wide-spread mucoid degeneration with cyst formation and mucous masses. The fetus died in one of the cases (Bergman et al., 1961). In 1985, Labarrerre et al. described three cases where the Wharton’s jelly was only present around the umbilical vein, leaving the umbilical arteries hanging free. In all three cases, the newborn died with meconium found either in the amniotic fluid or in the respiratory tract (Labarrere et al., 1985). In 2007, Kulkarni et al. reported a case of absent Wharton’s jelly associated with persistent vitello-intestinal duct (Kulkarni et al., 2007) and in 2020, Murphy et al. described a case of Wharton’s jelly absence around all three umbilical vessels with fetal bradycardia, and meconium staining of all three umbilical vessels and of the placenta. The newborn survived but suffered from meconium aspiration syndrome (Murphy et al., 2020).

In humans and other mammals, while the umbilical arteries occlude rapidly after delivery of the newborn, the umbilical vein remains patent longer to allow placental infusion. Indeed, while the umbilical arteries have a bilaminar structure (Meyer et al., 1978) composed of an outer and an inner tunica media, with no elastic structure, no distinct inner layer is observed in the vein. Even though smooth muscle cells are observed equally within both tunica media of the arteries and of the vein, the latter contains fewer layers of smooth muscle cells. Thus, the umbilical arteries have a much thicker tunica media than the vein. In addition, the outer tunica media of umbilical arteries includes more contractile smooth muscle cells compared to the inner tunica media and the vein, allowing vasoconstriction of umbilical arteries through folding of the inner layer for lumen occlusion at birth. Furthermore, the proteoglycan composition of the extracellular matrix of the umbilical vessels plays a role in vasoconstriction. While the inner tunica media of the umbilical arteries is rich in proteoglycans, the expression of these proteoglycans is very weak in the arterial outer tunica media and the venous tunica media. This composition results from the expression of proteoglycanases called ADAMTS. ADAMTS are highly expressed in the venous tunica media, specifically prior to parturition, and in the arterial outer tunica media. ADAMTS proteolyzes the proteoglycans, thus reducing its level in the vein and the outer tunica media of the arteries. The low level of ADAMTS allows the maintenance of proteoglycans in the inner tunica media of the arteries, leading to rapid arterial occlusion at birth (Nandadasa et al., 2015; 2020).

#### 3.2.3 Piezo1 as blood flow sensor in the human umbilical vasculature

Piezo proteins are essential determinants of vascular structures that act as sensors of blood flow through their dependence on shear stress-evoked ionic current and calcium influx (Figure 3D) (Li et al., 2014a; Ranade et al., 2014; Xiao et al., 2023). The importance of Piezo1 in the development of human umbilical veins was demonstrated notably through experiments in cultured HUVECs. Indeed, depletion of Piezo1 led to suppressed migration of endothelial cells, thus inhibiting umbilical vein tube formation (Li et al., 2014a). Li and colleagues performed mass spectrometry to identify proteins affected by Piezo1 depletion in static and shear stress conditions. Results showed reduction of endothelial nitric oxide synthase (eNOS) upon Piezo1 depletion in both static and shear stress conditions. In addition, phosphorylation of eNOS at serine 1,177 by VEGF, which enhances eNOS activity, was abolished in static endothelial cells depleted of Piezo1. Endothelial cell migration was similarly affected by Piezo1 depletion, eNOS depletion, and NOS inhibition. The defective alignment observed in Piezo1 depleted HUVECs was not due to an effect on NO/eNOS, but rather on cytoskeletal regulation mediated through activation of calpain-mediated proteolytic cleavage of actin cytoskeleton and focal adhesion proteins (Li et al., 2014a).

In addition to the effect of shear stress on Piezo1 in the apical membrane of endothelial cells, Piezo1 also senses membrane tension at the junctional cell side through the adherens junction force sensing machinery. In this case, Piezo1 activity is regulated by PECAM1 in endothelial cells. In response to membrane tension, PECAM1 inhibits Piezo1 activity. The C-terminal structure of PECAM1, particularly Y713, interacts with N-terminal regions of Piezo1 to reduce its mechanical sensitivity. Thus, an increased abundance of PECAM1 reduces Piezo1 channel function and decreases calcium influx in endothelial cells (Figure 3D) (Chuntharpursat-Bon et al., 2023).

## 4 Discussion

The mouse is often used as a model to study human development. Due to its transient and fragile nature, the human yolk sac is challenging to study. The mouse yolk sac, however, remains throughout gestation; it has a similar histological makeup, and cells that cover equivalent functions (Martinelli et al., 2023), thereby offering a great opportunity to study early vascular and blood cell development.

There is still relatively little known on the development of the umbilical vessels. While blood flow velocity was measured in several species, including mouse and human (Table 3) (Mu and Adamson, 2006; Osol and Mandala, 2009; Linask et al., 2014; Zhou et al., 2014; Downs, 2022; Barbieri et al., 2023), a thorough investigation of hemodynamic forces and their impact on vascular development and remodeling in the umbilical cord and placenta is yet to be performed. To study vascular development quantitatively, one can measure vessel and blood flow parameters such as the pulsatility index and refractive index, as well as end-diastolic velocity, end-systolic velocity, and peak systolic velocity (Linask et al., 2014), among others. Various methods to study flow *in vivo* can be used to investigate its role in vascular development, remodeling and maturation (Daems et al., 2020).

Although there are differences between the murine and human umbilical cord and placenta, the mouse is a useful model (Soares et al., 2017; 2018; Woods et al., 2018). Blood flow in the umbilical cord only occurs after chorio-allantoic fusion and after the umbilical artery connects to the vessel of confluence (Linask et al., 2014). Therefore, the initial vascular development of the umbilical cord is likely independent from hemodynamic factors. Another non mechanical driver for placenta development is hypoxia (Soares et al., 2017). Several studies have shown the effect of fluid shear stress on vascular remodeling via NO production in mouse and human endothelial cells (Tao et al., 2006; Lucitti et al., 2007; Lu and Kassab, 2011; Li et al., 2014a; Wang et al., 2016; Albarrán-Juárez et al., 2018; Xiao et al., 2023). Shear stress activates the endothelial mechanosensitive cation channel Piezo1 (Xiao et al., 2023), increasing the intracellular Ca^2+^ concentration and releasing ATP. ATP-release leads to the activation of the Gq/G_11_-coupled purinergic P2Y2 receptor on endothelial cells (Wang et al., 2016; Morley et al., 2019). The increased intracellular concentration of Ca^2+^ upregulates *eNOS* mRNA expression in a dose-dependent manner through Ca^2+^/calmodulin, as well as phosphorylation of eNOS at Ser1176 and Ser320 sites by PKA or PI3K/AKT via the mechanosensory complex, consisting of PECAM1, VE-cadherin and VEGR2/VEGFR3 (Figure 3D) (Dimmeler et al., 1999; Balligand et al., 2009; Wang et al., 2016; Morley et al., 2019). Intracellular eNOS is subsequently translocated to the endothelial cell membrane (Lucitti et al., 2007) and increases the production of the key vasodilator NO. Overall, this downstream pathway results in vascular relaxation (Tao et al., 2006; Xiao et al., 2023). NO plays a critical role in systemic blood pressure regulation (Wang et al., 2016) and is required for normal vascular remodeling (Lucitti et al., 2007).

The development of the embryonic vascular system relies on the morphogenesis of the extraembryonic tissues. The study of the early steps of embryogenesis is crucial to understand the causes of pathologies of pregnancy, from first trimester loss to later anomalies. However, ethical and technical challenges are limiting factors. In human, negative pregnancy outcomes have been associated to ectopic insertion of the umbilical cord into the chorion (Jauniaux et al., 2020). Malperfusion of the uterus and/or placenta alter smooth muscle cell proliferation. A smooth muscle cell excess surrounding the placental arteries increases the umbilical resistance to flow, elevating the afterload on the developing heart (Burton and Jauniaux, 2018a). In addition, while hypoxia is a driving force for placental development, it can result from placental defects (Soares et al., 2017; 2018). Single umbilical artery, a condition where only one umbilical artery is present in human instead of the usual two, is most often benign but is a risk factor for defective nutrient and waste exchange between the mother and fetus and can lead to intrauterine growth restriction. Furthermore, single umbilical artery may cause hemodynamic changes that increase the afterload on the developing heart, resulting in right ventricular defects (Rossant and Cross, 2001; Burton and Jauniaux, 2018a; Woods et al., 2018; Li et al., 2019; Ebbing et al., 2020). The mouse has proven a valuable model to study both normal and aberrant placenta development (Rossant and Cross, 2001; Perez-Garcia et al., 2018; Woods et al., 2018).

The recent advances in mouse and human stem cell-based embryo models and organoids offer new opportunities to study the formation of embryonic but also extraembryonic structures and are likely to reduce the need for human tissues or laboratory animals. Recently, an mouse embryo model was created by combining embryonic, trophoblastic, and extraembryonic endoderm stem cells (Amadei et al., 2022). These embryoids develop from the first stages of embryogenesis (gastrulation, neurulation, and early organogenesis) up to a stage resembling E8.5. While placentation cannot be fully investigated, allantois- and YS-like structures are present. Furthermore, a beating heart develops even though no looping of the heart tube was observed. While a vascular system was not described, Runx1^+^ structures that are similar to blood islands are formed in the YS-like tissues and at the base of the structure that resembles the allantois. A human stem cell-based embryo model was also recently created from naïve pluripotent stem cells that act as totipotent stem cells (Oldak et al., 2023). Those initially present the epiblast and hypoblast lineages, and form the amniotic sac and YS cavities but do not go through a blastocyst stage. These self-assembling embryoids imitate the main developmental structures of natural human embryos until 13–14 days post-fertilization.

Although those stem cells-based models are not suitable to study extraembryonic circulation yet, it is likely that further research may offer the opportunity to uncover flow-related mechanisms of extraembryonic vascular development, notably through association with organoids reproducing the endometrium. Combining experiments on mouse embryos and stem cell-based models would be a powerful and ethical way to improve our knowledge of the establishment of the maternal/fetal interface.

## References

[B1] Aitsebaomo J. Portbury A. L. Schisler J. C. Patterson C. (2008). Brothers and sisters: molecular insights into arterial-venous heterogeneity. Circ. Res. 103, 929–939. 10.1161/CIRCRESAHA.108.184937 18948631 PMC2760069

[B2] Albarrán-Juárez J. Iring A. Wang S. Joseph S. Grimm M. Strilic B. (2018). Piezo1 and Gq/G11 promote endothelial inflammation depending on flow pattern and integrin activation. J. Exp. Med. 215, 2655–2672. 10.1084/jem.20180483 30194266 PMC6170174

[B3] Amadei G. Handford C. E. Qiu C. De Jonghe J. Greenfeld H. Tran M. (2022). Embryo model completes gastrulation to neurulation and organogenesis. Nature 610, 143–153. 10.1038/s41586-022-05246-3 36007540 PMC9534772

[B4] Arora R. Papaioannou V. E. (2012). The murine allantois: a model system for the study of blood vessel formation. Blood 120, 2562–2572. 10.1182/blood-2012-03-390070 22855605 PMC3460680

[B5] Arthur J. S. C. Elce J. S. Hegadorn C. Williams K. Greer P. A. (2000). Disruption of the murine calpain small subunit gene, Capn4: calpain is essential for embryonic development but not for cell growth and division. Mol. Cell. Biol. 20, 4474–4481. 10.1128/MCB.20.12.4474-4481.2000 10825211 PMC85815

[B6] Baeyens N. Schwartz M. A. (2016). Biomechanics of vascular mechanosensation and remodeling. Mol. Biol. Cell 27, 7–11. 10.1091/mbc.E14-11-1522 26715421 PMC4694763

[B7] Balligand J.-L. Feron O. Dessy C. (2009). eNOS activation by physical forces: from short-term regulation of contraction to chronic remodeling of cardiovascular tissues. Physiol. Rev. 89, 481–534. 10.1152/physrev.00042.2007 19342613

[B8] Barbieri M. Di Martino D. D. Ferrazzi E. M. Stampalija T. (2023). Umbilical vein blood flow: state-of-the-art. J. Clin. Ultrasound 51, 318–325. 10.1002/jcu.23412 36785504

[B9] Basta M. Lipsett B. J. (2023). Anatomy, abdomen and pelvis: umbilical cord. Treasure Island, FL: StatPearls Publishing Available at: https://www.ncbi.nlm.nih.gov/books/NBK557389/ .

[B10] Bergman P. Lundin P. Malmström T. (1961). Mucoid degeneration of Wharton’s jelly. An umbilical cord anomaly threatening fœtal life. Acta Obstet. Gynecol. Scand. 40, 372–378. 10.3109/00016346109159935 13867744

[B11] Bernstein I. M. Ziegler W. F. Leavitt T. Badger G. J. (2002). Uterine artery hemodynamic adaptations through the menstrual cycle into early pregnancy. Obstet. Gynecol. 99, 620–624. 10.1016/s0029-7844(01)01787-2 12039123

[B133] Blanco-Elices C. Chato-Astrain J. González-González A. Sánchez-Porras D. Carriel V. Fernández-Valadés R. (2022). Histological profiling of the human umbilical cord: a potential alternative cell source in tissue engineering. J. Pers. Med. 12. 10.3390/jpm12040648 PMC9028794 35455764

[B12] Burton G. J. Jauniaux E. (2018a). Development of the human placenta and fetal heart: synergic or independent? Front. Physiol. 9, 373–410. 10.3389/fphys.2018.00373 29706899 PMC5906582

[B13] Burton G. J. Jauniaux E. (2018b). Pathophysiology of placental-derived fetal growth restriction. Am. J. Obstet. Gynecol. 218, S745–S761. 10.1016/j.ajog.2017.11.577 29422210

[B14] Burton G. J. Jauniaux E. Charnock-Jones D. S. (2010). The influence of the intrauterine environment on human placental development. Int. J. Dev. Biol. 54, 303–312. 10.1387/ijdb.082764gb 19757391

[B15] Campinho P. Vilfan A. Vermot J. (2020). Blood flow forces in shaping the vascular system: a focus on endothelial cell behavior. Front. Physiol. 11, 552–612. 10.3389/fphys.2020.00552 32581842 PMC7291788

[B16] Carvalho R. L. C. Itoh F. Goumans M.-J. Lebrin F. Kato M. Takahashi S. (2007). Compensatory signalling induced in the yolk sac vasculature by deletion of TGFbeta receptors in mice. J. Cell Sci. 120, 4269–4277. 10.1242/jcs.013169 18029401

[B17] Chazara O. Xiong S. Moffett A. (2011). Maternal KIR and fetal HLA-C: a fine balance. J. Leukoc. Biol. 90, 703–716. 10.1189/jlb.0511227 21873457

[B18] Chong D. C. Koo Y. Xu K. Fu S. Cleaver O. (2011). Stepwise arteriovenous fate acquisition during mammalian vasculogenesis. Dev. Dyn. 240, 2153–2165. 10.1002/dvdy.22706 21793101 PMC3192916

[B19] Chuntharpursat-Bon E. Povstyan O. V. Ludlow M. J. Carrier D. J. Debant M. Shi J. (2023). PIEZO1 and PECAM1 interact at cell-cell junctions and partner in endothelial force sensing. Commun. Biol. 6, 358. 10.1038/s42003-023-04706-4 37005489 PMC10067937

[B20] Collart C. Ciccarelli A. Ivanovitch K. Rosewell I. Kumar S. Kelly G. (2021). The migratory pathways of the cells that form the endocardium, dorsal aortae, and head vasculature in the mouse embryo. BMC Dev. Biol. 21, 8–18. 10.1186/s12861-021-00239-3 33752600 PMC7986287

[B21] Coon B. G. Baeyens N. Han J. Budatha M. Ross T. D. Fang J. S. (2015). Intramembrane binding of VE-cadherin to VEGFR2 and VEGFR3 assembles the endothelial mechanosensory complex. J. Cell Biol. 208, 975–986. 10.1083/jcb.201408103 25800053 PMC4384728

[B22] Culver J. C. Dickinson M. E. (2010). The effects of hemodynamic force on embryonic development. Microcirculation 17, 164–178. 10.1111/j.1549-8719.2010.00025.x 20374481 PMC2927969

[B23] Daems M. Peacock H. M. Jones E. A. V. (2020). Fluid flow as a driver of embryonic morphogenesis. Development 147, dev185579. 10.1242/dev.185579 32769200

[B24] dela Paz N. G. Walshe T. E. Leach L. L. Saint-Geniez M. D’Amore P. A. (2012). Role of shear-stress-induced VEGF expression in endothelial cell survival. J. Cell Sci. 125, 831–843. 10.1242/jcs.084301 22399811 PMC3311927

[B25] Diehl K. H. Hull R. Morton D. Pfister R. Rabemampianina Y. Smith D. (2001). A good practice guide to the administration of substances and removal of blood, including routes and volumes. J. Appl. Toxicol. 21, 15–23. 10.1002/jat.727 11180276

[B26] Dimmeler S. Fleming I. Fisslthaler B. Hermann C. Busse R. Zeiher A. M. (1999). Activation of nitric oxide synthase in endothelial cells by Akt-dependent phosphorylation. Nature 399, 601–605. 10.1038/21224 10376603

[B27] Downs K. M. (2006). *In vitro* methods for studying vascularization of the murine allantois and allantoic union with the chorion. Methods Mol. Med. 121, 241–272. 10.1385/1-59259-983-4:239 16251748

[B28] Downs K. M. (2022). The mouse allantois: new insights at the embryonic-extraembryonic interface. Philos. Trans. R. Soc. B Biol. Sci. 377, 20210251. 10.1098/rstb.2021.0251 PMC9574631 36252214

[B29] Downs K. M. Gifford S. Blahnik M. Gardner R. L. (1998). Vascularization in the murine allantois occurs by vasculogenesis without accompanying erythropoiesis. Development 125, 4507–4520. 10.1242/dev.125.22.4507 9778509

[B30] Downs K. M. Harmann C. (1997). Developmental potency of the murine allantois. Development 124, 2769–2780. 10.1242/dev.124.14.2769 9226448

[B31] Downs K. M. Rodriguez A. M. (2020). The mouse fetal-placental arterial connection: a paradigm involving the primitive streak and visceral endoderm with implications for human development. WIREs Dev. Biol. 9, 3622–e430. 10.1002/wdev.362 31622045

[B32] Downs K. M. Temkin R. Gifford S. McHugh J. (2001). Study of the murine allantois by allantoic explants. Dev. Biol. 233, 347–364. 10.1006/dbio.2001.0227 11336500

[B33] Ebbing C. Kessler J. Moster D. Rasmussen S. (2020). Isolated single umbilical artery and the risk of adverse perinatal outcome and third stage of labor complications: a population-based study. Acta Obstet. Gynecol. Scand. 99, 374–380. 10.1111/aogs.13747 31603530

[B34] Egorova A. D. DeRuiter M. C. De Boer H. C. Van De Pas S. Gittenberger-De Groot A. C. Van Zonneveld A. J. (2012). Endothelial colony-forming cells show a mature transcriptional response to shear stress. Vitr. Cell. Dev. Biol. - Anim. 48, 21–29. 10.1007/s11626-011-9470-z 22101679

[B35] Elmore S. A. Cochran R. Z. Bolon B. Lubeck B. Mahler B. Sabio D. (2022). Histology atlas of the developing mouse placenta. Toxicol. Pathol. 50, 60–117. 10.1177/01926233211042270 34872401 PMC8678285

[B36] Erlebacher A. (2013). Immunology of the maternal-fetal interface. Annu. Rev. Immunol. 31, 387–411. 10.1146/annurev-immunol-032712-100003 23298207

[B37] Fåhræus R. Lindqvist T. (1931). THE VISCOSITY OF THE BLOOD IN NARROW CAPILLARY TUBES. Am. J. Physiol. Content 96, 562–568. 10.1152/ajplegacy.1931.96.3.562

[B38] Farina A. Rosso F. Fasano A. (2021). A continuum mechanics model for the Fåhræus-Lindqvist effect. J. Biol. Phys. 47, 253–270. 10.1007/s10867-021-09575-8 34218404 PMC8452817

[B39] Ferdous A. Morris J. Abedin M. J. Collins S. Richardson J. A. Hill J. A. (2011). Forkhead factor FoxO1 is essential for placental morphogenesis in the developing embryo. Proc. Natl. Acad. Sci. 108, 16307–16312. 10.1073/pnas.1107341108 21930913 PMC3182720

[B40] Ferkowicz M. J. Starr M. Xie X. Li W. Johnson S. A. Shelley W. C. (2003). CD41 expression defines the onset of primitive and definitive hematopoiesis in the murine embryo. Development 130, 4393–4403. 10.1242/dev.00632 12900455

[B41] Forbes T. R. Taku E. (1975). Vein size in intact and hysterectomized mice during the estrous cycle and pregnancy. Anat. Rec. 182, 61–65. 10.1002/ar.1091820107 1155791

[B42] Garcia M. D. Larina I. V. (2014). Vascular development and hemodynamic force in the mouse yolk sac. Front. Physiol. 5 AUG, 308–310. 10.3389/fphys.2014.00308 25191274 PMC4138559

[B43] Ge J. Li W. Zhao Q. Li N. Chen M. Zhi P. (2015). Architecture of the mammalian mechanosensitive Piezo1 channel. Nature 527, 64–69. 10.1038/nature15247 26390154

[B44] Graupera M. Guillermet-Guibert J. Foukas L. C. Phng L. K. Cain R. J. Salpekar A. (2008). Angiogenesis selectively requires the p110alpha isoform of PI3K to control endothelial cell migration. Nature 453, 662–666. 10.1038/nature06892 18449193

[B45] Gulbis B. Jauniaux E. Cotton F. Stordeur P. (1998). Protein and enzyme patterns in the fluid cavities of the first trimester gestational sac: relevance to the absorptive role of secondary yolk sac. Mol. Hum. Reprod. 4, 857–862. 10.1093/molehr/4.9.857 9783845

[B46] Guo Y. R. MacKinnon R. (2017). Structure-based membrane dome mechanism for Piezo mechanosensitivity. Elife 6, 336600–e33719. 10.7554/eLife.33660 PMC5788504 29231809

[B47] Hadamek K. Keller A. Gohla A. (2018). Dissection and explant culture of murine allantois for the *in vitro* analysis of allantoic attachment. J. Vis. Exp. 2018, 56712–56718. 10.3791/56712 PMC5908651 29364244

[B48] Hemberger M. Hanna C. W. Dean W. (2020). Mechanisms of early placental development in mouse and humans. Nat. Rev. Genet. 21, 27–43. 10.1038/s41576-019-0169-4 31534202

[B49] Herzog Y. Guttmann-Raviv N. Neufeld G. (2005). Segregation of arterial and venous markers in subpopulations of blood islands before vessel formation. Dev. Dyn. 232, 1047–1055. 10.1002/dvdy.20257 15739224

[B50] Hoefer I. E. den Adel B. Daemen M. J. A. P. (2013). Biomechanical factors as triggers of vascular growth. Cardiovasc. Res. 99, 276–283. 10.1093/cvr/cvt089 23580605

[B51] Hou W. Sarikaya D. P. Jerome-Majewska L. A. (2016). *Ex vivo* culture of pre-placental tissues reveals that the allantois is required for maintained expression of Gcm1 and Tpbpα. Placenta 47, 12–23. 10.1016/j.placenta.2016.08.091 27780534

[B52] Huang C. Sheikh F. Hollander M. Cai C. Becker D. Chu P.-H. (2003). Embryonic atrial function is essential for mouse embryogenesis, cardiac morphogenesis and angiogenesis. Development 130, 6111–6119. 10.1242/dev.00831 14573518

[B53] Inman K. E. Downs K. M. (2007). The murine allantois: emerging paradigms in development of the mammalian umbilical cord and its relation to the fetus. Genesis 45, 237–258. 10.1002/dvg.20281 17440924

[B54] Jauniaux E. Hempstock J. Greenwold N. Burton G. J. (2003). Trophoblastic oxidative stress in relation to temporal and regional differences in maternal placental blood flow in normal and abnormal early pregnancies. Am. J. Pathol. 162, 115–125. 10.1016/S0002-9440(10)63803-5 12507895 PMC1851128

[B55] Jauniaux E. Jurkovic D. Henriet Y. Rodesch F. Hustin J. (1991). Development of the secondary human yolk sac: correlation of sonographic and anatomical features. Hum. Reprod. 6, 1160–1166. 10.1093/oxfordjournals.humrep.a137503 1806578

[B56] Jauniaux E. Moffett A. Burton G. J. (2020). Placental implantation disorders. Obstet. Gynecol. Clin. North Am. 47, 117–132. 10.1016/j.ogc.2019.10.002 32008663

[B57] Jones E. A. V. Baron M. H. Fraser S. E. Dickinson M. E. (2004). Measuring hemodynamic changes during mammalian development. Am. J. Physiol. - Hear. Circ. Physiol. 287, 1561–1569. 10.1152/ajpheart.00081.2004 15155254

[B58] Jones E. A. V. Le Noble F. Eichmann A. (2006). What determines blood vessel structure? Genetic prespecification vs. hemodynamics. Physiology 21, 388–395. 10.1152/physiol.00020.2006 17119151

[B59] Jones E. A. V. Yuan L. Breant C. Watts R. J. Eichmann A. (2008). Separating genetic and hemodynamic defects in neuropilin 1 knockout embryos. Development 135, 2479–2488. 10.1242/dev.014902 18550715

[B60] Jordan H. E. (1910). A further study of the human umbilical vesicle. Anat. Rec. 4, 341–353. 10.1002/ar.1090040903

[B61] Kingdom J. Huppertz B. Seaward G. Kaufmann P. (2000). Development of the placental villous tree and its consequences for fetal growth. Eur. J. Obstet. Gynecol. Reprod. Biol. 92, 35–43. 10.1016/S0301-2115(00)00423-1 10986432

[B62] Krebs L. T. Shutter J. R. Tanigaki K. Honjo T. Stark K. L. Gridley T. (2004). Haploinsufficient lethality and formation of arteriovenous malformations in Notch pathway mutants. Genes. Dev. 18, 2469–2473. 10.1101/gad.1239204 15466160 PMC529533

[B63] Kulkarni M. L. Matadh P. S. Ashok C. Pradeep N. Avinash T. Kulkarni A. M. (2007). Absence of Wharton’s jelly around the umbilical arteries. Indian J. Pediatr. 74, 787–789. 10.1007/s12098-007-0142-7 17785908

[B64] Labarrere C. Sebastiani M. Siminovich M. Torassa E. Althabe O. (1985). Absence of Wharton’s jelly around the umbilical arteries: an unusual cause of perinatal mortality. Placenta 6, 555–559. 10.1016/S0143-4004(85)80010-2 3836403

[B65] Lebart M. Benyamin Y. (2006). Calpain involvement in the remodeling of cytoskeletal anchorage complexes. FEBS J. 273, 3415–3426. 10.1111/j.1742-4658.2006.05350.x 16884487

[B66] le Noble F. Fleury V. Pries A. Corvol P. Eichmann A. Reneman R. S. (2005). Control of arterial branching morphogenesis in embryogenesis: go with the flow. Cardiovasc. Res. 65, 619–628. 10.1016/j.cardiores.2004.09.018 15664388

[B67] le Noble F. Moyon D. Pardanaud L. Yuan L. Djonov V. Matthijsen R. (2004). Flow regulates arterial-venous differentiation in the chick embryo yolk sac. Development 131, 361–375. 10.1242/dev.00929 14681188

[B68] Li J. Hou B. Tumova S. Muraki K. Bruns A. Ludlow M. J. (2014a). Piezo1 integration of vascular architecture with physiological force. Nature 515, 279–282. 10.1038/nature13701 25119035 PMC4230887

[B69] Li R. Beebe T. Jen N. Yu F. Takabe W. Harrison M. (2014b). Shear stress–activated wnt-angiopoietin-2 signaling recapitulates vascular repair in zebrafish embryos. Arterioscler. Thromb. Vasc. Biol. 34, 2268–2275. 10.1161/ATVBAHA.114.303345 25147335 PMC4169303

[B70] Li T. Nie F. Li Z. Wang Y. Li Q. (2019). Evaluation of right ventricular function in fetuses with isolated single umbilical artery using spatiotemporal image correlation M-mode. Cardiovasc. Ultrasound 17, 14. 10.1186/s12947-019-0164-0 31325956 PMC6642479

[B71] Linask K. K. Han M. Bravo-Valenzuela N. J. M. (2014). Changes in vitelline and utero-placental hemodynamics: implications for cardiovascular development. Front. Physiol. 5, 390–412. 10.3389/fphys.2014.00390 25426076 PMC4227466

[B72] Li-Villarreal N. Wong R. L. Y. Garcia M. D. Udan R. S. Poché R. A. Rasmussen T. L. (2022). FOXO1 represses sprouty 2 and sprouty 4 expression to promote arterial specification and vascular remodeling in the mouse yolk sac. Development 149, dev200131. 10.1242/dev.200131 35297995 PMC8995087

[B73] Lu D. Kassab G. S. (2011). Role of shear stress and stretch in vascular mechanobiology. J. R. Soc. Interface 8, 1379–1385. 10.1098/rsif.2011.0177 21733876 PMC3163429

[B74] Lu J. Wu W. Xin Q. Zhou C. Wang J. Ni Z. (2019). Spatiotemporal coordination of trophoblast and allantoic Rbpj signaling directs normal placental morphogenesis. Cell Death Dis. 10, 438–514. 10.1038/s41419-019-1683-1 31165749 PMC6549187

[B75] Lucitti J. L. Jones E. A. V. Huang C. Chen J. Fraser S. E. Dickinson M. E. (2007). Vascular remodeling of the mouse yolk sac requires hemodynamic force. Development 134, 3317–3326. 10.1242/dev.02883 17720695 PMC4260474

[B76] Luckett W. P. (1978). Origin and differentiation of the yolk sac and extraembryonic mesoderm in presomite human and rhesus monkey embryos. Am. J. Anat. 152, 59–97. 10.1002/aja.1001520106 98035

[B77] Luse M. A. Jackson M. G. Juśkiewicz Z. J. Isakson B. E. (2023). Physiological functions of caveolae in endothelium. Curr. Opin. Physiol. 35, 100701. 10.1016/j.cophys.2023.100701 37873030 PMC10588508

[B78] Luu V. Z. Chowdhury B. Al-Omran M. Hess D. A. Verma S. (2018). Role of endothelial primary cilia as fluid mechanosensors on vascular health. Atherosclerosis 275, 196–204. 10.1016/j.atherosclerosis.2018.06.818 29945035

[B79] Mandalà M. (2020). Influence of estrogens on uterine vascular adaptation in normal and preeclamptic pregnancies. Int. J. Mol. Sci. 21, 2592. 10.3390/ijms21072592 32276444 PMC7177259

[B80] Mantoni M. Pedersen J. F. (1979). Ultrasound visualization of the human yolk sac. J. Clin. Ultrasound 7, 459–460. 10.1002/jcu.1870070608 118186

[B81] Martinelli L. M. Carucci A. Payano V. J. H. Connor K. L. Bloise E. (2023). Translational comparison of the human and mouse yolk sac development and function. Reprod. Sci. 30, 41–53. 10.1007/s43032-022-00872-8 35137348

[B82] McGrath K. E. Koniski A. D. Malik J. Palis J. (2003). Circulation is established in a stepwise pattern in the mammalian embryo. Blood 101, 1669–1676. 10.1182/blood-2002-08-2531 12406884

[B83] McHugh B. J. Buttery R. Lad Y. Banks S. Haslett C. Sethi T. (2010). Integrin activation by Fam38A uses a novel mechanism of R-Ras targeting to the endoplasmic reticulum. J. Cell Sci. 123, 51–61. 10.1242/jcs.056424 20016066 PMC2794710

[B84] Mehta V. Pang K.-L. Rozbesky D. Nather K. Keen A. Lachowski D. (2020). The guidance receptor plexin D1 is a mechanosensor in endothelial cells. Nature 578, 290–295. 10.1038/s41586-020-1979-4 32025034 PMC7025890

[B85] Meyer W. W. Rumpelt H. J. Yao A. C. Lind J. (1978). Structure and closure mechanism of the human umbilical artery. Eur. J. Pediatr. 128, 247–259. 10.1007/BF00445610 668732

[B86] Miyazaki T. Honda K. Ohata H. (2007). Requirement of Ca 2+ influx- and phosphatidylinositol 3-kinase-mediated m-calpain activity for shear stress-induced endothelial cell polarity. Am. J. Physiol. Physiol. 293, C1216–C1225. 10.1152/ajpcell.00083.2007 17596297

[B87] Morley L. C. Beech D. J. Walker J. J. Simpson N. A. B. (2019). Emerging concepts of shear stress in placental development and function. Mol. Hum. Reprod. 25, 329–339. 10.1093/molehr/gaz018 30931481 PMC6554190

[B88] Mu J. Adamson S. L. (2006). Developmental changes in hemodynamics of uterine artery, utero- and umbilicoplacental, and vitelline circulations in mouse throughout gestation. Am. J. Physiol. - Hear. Circ. Physiol. 291, 1421–1428. 10.1152/ajpheart.00031.2006 16603699

[B89] Murphy S. J. Deegan N. O’Leary B. D. McParland P. (2020). Absence of Wharton’s jelly. BMJ Case Rep. 13, e237222–e237223. 10.1136/bcr-2020-237222 PMC7705544 33257379

[B90] Myatt L. (1992). Control of vascular resistance in the human placenta. Placenta 13, 329–341. 10.1016/0143-4004(92)90057-Z 1438081

[B91] Nandadasa S. Nelson C. M. Apte S. S. (2015). ADAMTS9-Mediated extracellular matrix dynamics regulates umbilical cord vascular smooth muscle differentiation and rotation. Cell Rep. 11, 1519–1528. 10.1016/j.celrep.2015.05.005 26027930 PMC4472575

[B92] Nandadasa S. Szafron J. M. Pathak V. Murtada S. Kraft C. M. O’donnell A. (2020). Vascular dimorphism ensured by regulated proteoglycan dynamics favors rapid umbilical artery closure at birth. Elife 9, 606833–e60730. 10.7554/ELIFE.60683 PMC7529456 32909945

[B93] Oldak B. Wildschutz E. Bondarenko V. Comar M. Y. Zhao C. Aguilera-Castrejon A. (2023). Complete human day 14 post-implantation embryo models from naive ES cells. Nature 622, 562–573. 10.1038/s41586-023-06604-5 37673118 PMC10584686

[B94] Osol G. Mandala M. (2009). Maternal uterine vascular remodeling during pregnancy. Physiology 24, 58–71. 10.1152/physiol.00033.2008 19196652 PMC2760472

[B95] Palis J. Yoder M. C. (2001). Yolk-sac hematopoiesis: the first blood cells of mouse and man. Exp. Hematol. 29, 927–936. 10.1016/S0301-472X(01)00669-5 11495698

[B96] Pang Y. Dong J. Thomas P. (2015). Progesterone increases nitric oxide synthesis in human vascular endothelial cells through activation of membrane progesterone receptor-α. Am. J. Physiol. Metab. 308, E899–E911. 10.1152/ajpendo.00527.2014 25805192

[B97] Panja S. Paria B. C. (2021). Development of the mouse placenta. Adv. Anat. Embryol. Cell Biol. 234, 205–221. 10.1007/978-3-030-77360-1_10 34694483 PMC9109784

[B98] Perez-Garcia V. Fineberg E. Wilson R. Murray A. Mazzeo C. I. Tudor C. (2018). Placentation defects are highly prevalent in embryonic lethal mouse mutants. Nature 555, 463–468. 10.1038/nature26002 29539633 PMC5866719

[B99] Ramesh S. Hariprasath S. Anandan G. Solomon Pj. Vijayakumar V. (2015). Single umbilical artery. J. Pharm. Bioallied Sci. 7, 83–S84. 10.4103/0975-7406.155815 PMC4439720 26015760

[B100] Ranade S. S. Qiu Z. Woo S. H. Hur S. S. Murthy S. E. Cahalan S. M. (2014). Piezo1, a mechanically activated ion channel, is required for vascular development in mice. Proc. Natl. Acad. Sci. U. S. A. 111, 10347–10352. 10.1073/pnas.1409233111 24958852 PMC4104881

[B101] Rashdan N. A. Lloyd P. G. (2015). Fluid shear stress upregulates placental growth factor in the vessel wall via NADPH oxidase 4. Am. J. Physiol. - Hear. Circ. Physiol. 309, H1655–H1666. 10.1152/ajpheart.00408.2015 PMC4666978 26408539

[B102] Rizzo V. Sung A. Oh P. Schnitzer J. E. (1998). Rapid mechanotransduction *in situ* at the luminal cell surface of vascular endothelium and its caveolae. J. Biol. Chem. 273, 26323–26329. 10.1074/jbc.273.41.26323 9756862

[B103] Rodriguez A. M. Downs K. M. (2017). Visceral endoderm and the primitive streak interact to build the fetal-placental interface of the mouse gastrula. Dev. Biol. 432, 98–124. 10.1016/j.ydbio.2017.08.026 28882402 PMC5980994

[B104] Rodriguez A. M. Jin D. X. Wolfe A. D. Mikedis M. M. Wierenga L. Hashmi M. P. (2017). Brachyury drives formation of a distinct vascular branchpoint critical for fetal-placental arterial union in the mouse gastrula. Dev. Biol. 425, 208–222. 10.1016/j.ydbio.2017.03.032 28389228 PMC5760991

[B105] Rossant J. Cross J. C. (2001). Placental development: lessons from mouse mutants. Nat. Rev. Genet. 2, 538–548. 10.1038/35080570 11433360

[B106] Saotome K. Murthy S. E. Kefauver J. M. Whitwam T. Patapoutian A. Ward A. B. (2018). Structure of the mechanically activated ion channel Piezo1. Nature 554, 481–486. 10.1038/nature25453 29261642 PMC6010196

[B107] Saw S. N. Dawn C. Biswas A. Mattar C. N. Z. Yap C. H. (2017). Characterization of the *in vivo* wall shear stress environment of human fetus umbilical arteries and veins. Biomech. Model. Mechanobiol. 16, 197–211. 10.1007/s10237-016-0810-5 27456489

[B108] Saw S. N. Poh Y. W. Chia D. Biswas A. Mattar C. N. Z. Yap C. H. (2018). Characterization of the hemodynamic wall shear stresses in human umbilical vessels from normal and intrauterine growth restricted pregnancies. Biomech. Model. Mechanobiol. 17, 1107–1117. 10.1007/s10237-018-1017-8 29691766

[B109] Shalaby F. Rossant J. Yamaguchi T. P. Gertsenstein M. Wu X. F. Breitman M. L. (1995). Failure of blood-island formation and vasculogenesis in Flk-1-deficient mice. Nature 376, 62–66. 10.1038/376062a0 7596435

[B110] Shay-Salit A. Shushy M. Wolfovitz E. Yahav H. Breviario F. Dejana E. (2002). VEGF receptor 2 and the adherens junction as a mechanical transducer in vascular endothelial cells. Proc. Natl. Acad. Sci. 99, 9462–9467. 10.1073/pnas.142224299 12080144 PMC123163

[B111] Shin H. Haga J. H. Kosawada T. Kimura K. Li Y. S. Chien S. (2019). Fine control of endothelial VEGFR-2 activation: caveolae as fluid shear stress shelters for membrane receptors. Biomech. Model. Mechanobiol. 18, 5–16. 10.1007/s10237-018-1063-2 30088112

[B132] Silini A. R. Di Pietro R. Lang-Olip I. Alviano F. Banerjee A. Basile M. (2020). Perinatal derivatives: Where do we stand? A roadmap of the human placenta and consensus for tissue and cell nomenclature. Front. Bioeng. Biotechnol 8, 1–33. 10.3389/fbioe.2020.610544 33392174 PMC7773933

[B112] Soares M. J. Iqbal K. Kozai K. (2017). Hypoxia and placental development. Birth Defects Res. 109, 1309–1329. 10.1002/bdr2.1135 29105383 PMC5743230

[B113] Soares M. J. Varberg K. M. Iqbal K. (2018). Hemochorial placentation: development, function, and adaptations. Biol. Reprod. 99, 196–211. 10.1093/biolre/ioy049 29481584 PMC6044390

[B114] Spurway J. Logan P. Pak S. (2012). The development, structure and blood flow within the umbilical cord with particular reference to the venous system. Australas. J. Ultrasound Med. 15, 97–102. 10.1002/j.2205-0140.2012.tb00013.x 28191152 PMC5025097

[B115] Strong T. H. Jarles D. L. Vega J. S. Feldman D. B. (1994). The umbilical coiling index. Am. J. Obstet. Gynecol. 170, 29–32. 10.1016/S0002-9378(94)70378-7 8296839

[B116] Tao J. Yang Z. Wang J.-M. Tu C. Pan S.-R. (2006). Effects of fluid shear stress on eNOS mRNA expression and NO production in human endothelial progenitor cells. Cardiology 106, 82–88. 10.1159/000092636 16612074

[B117] Tarbell J. M. Pahakis M. Y. (2006). Mechanotransduction and the glycocalyx. J. Intern. Med. 259, 339–350. 10.1111/j.1365-2796.2006.01620.x 16594902

[B118] Tavian M. Péault B. (2005). The changing cellular environments of hematopoiesis in human development *in utero* . Exp. Hematol. 33, 1062–1069. 10.1016/j.exphem.2005.06.025 16140155

[B119] Tzima E. Irani-Tehrani M. Kiosses W. B. Dejana E. Schultz D. A. Engelhardt B. (2005). A mechanosensory complex that mediates the endothelial cell response to fluid shear stress. Nature 437, 426–431. 10.1038/nature03952 16163360

[B120] Udan R. S. Culver J. C. Dickinson M. E. (2013a). Understanding vascular development. WIREs Dev. Biol. 2, 327–346. 10.1002/wdev.91 PMC4146572 23799579

[B121] Udan R. S. Vadakkan T. J. Dickinson M. E. (2013b). Dynamic responses of endothelial cells to changes in blood flow during vascular remodeling of the mouse yolk sac. Development 140, 4041–4050. 10.1242/dev.096255 24004946 PMC3775417

[B122] van der Heijden O. W. H. Essers Y. P. G. Fazzi G. Peeters L. L. H. De Mey J. G. R. van Eys G. J. J. M. (2005). Uterine artery remodeling and reproductive performance are impaired in endothelial nitric oxide synthase-deficient mice. Biol. Reprod. 72, 1161–1168. 10.1095/biolreprod.104.033985 15659709

[B123] Wang S. Chennupati R. Kaur H. Iring A. Wettschureck N. Offermanns S. (2016). Endothelial cation channel PIEZO1 controls blood pressure by mediating flow-induced ATP release. J. Clin. Invest. 126, 4527–4536. 10.1172/JCI87343 27797339 PMC5127677

[B124] Watson E. D. Cross J. C. (2005). Development of structures and transport functions in the mouse placenta. Physiology 20, 180–193. 10.1152/physiol.00001.2005 15888575

[B125] Weissman A. Jakobi P. Bronshtein M. Goldstein I. (1994). Sonographic measurements of the umbilical cord and vessels during normal pregnancies. J. Ultrasound Med. 13, 11–14. 10.7863/jum.1994.13.1.11 7636947

[B126] Woods L. Perez-Garcia V. Hemberger M. (2018). Regulation of placental development and its impact on fetal growth—new insights from mouse models. Front. Endocrinol. (Lausanne). 9, 570–618. 10.3389/fendo.2018.00570 30319550 PMC6170611

[B127] Wragg J. W. Durant S. McGettrick H. M. Sample K. M. Egginton S. Bicknell R. (2014). Shear stress regulated gene expression and angiogenesis in vascular endothelium. Microcirculation 21, 290–300. 10.1111/micc.12119 24471792

[B128] Xiao R. Liu J. Shawn Xu X. Z. (2023). Mechanosensitive GPCRs and ion channels in shear stress sensing. Curr. Opin. Cell Biol. 84, 102216. 10.1016/j.ceb.2023.102216 37595342 PMC10528224

[B129] Zhang J. Chen Z. Smith G. N. Croy B. A. (2011). Natural killer cell-triggered vascular transformation: maternal care before birth? Cell. Mol. Immunol. 8, 1–11. 10.1038/cmi.2010.38 20711229 PMC3079746

[B130] Zhao Q. Zhou H. Chi S. Wang Y. Wang J. Geng J. (2018). Structure and mechanogating mechanism of the Piezo1 channel. Nature 554, 487–492. 10.1038/nature25743 29469092

[B131] Zhou Y.-Q. Cahill L. S. Wong M. D. Seed M. Macgowan C. K. Sled J. G. (2014). Assessment of flow distribution in the mouse fetal circulation at late gestation by high-frequency Doppler ultrasound. Physiol. Genomics 46, 602–614. 10.1152/physiolgenomics.00049.2014 24963005

